# Caffeine does not cause override of the G2/M block induced by UVc or gamma radiation in normal human skin fibroblasts

**DOI:** 10.1054/bjoc.2000.1259

**Published:** 2000-07-03

**Authors:** G Deplanque, F Vincent, M C M Mah-Becherel, J-P Cazenave, J-P Bergerat, C Klein-Soyer

**Affiliations:** Laboratoire d’Oncologie Moléculaire, Institut de Recherche contre les Cancers de I’Appareil Digestif, Hôpitaux Universitaires de Strasbourg, BP 426, Strasbourg, 67091, France; INSERM U. 311, Etablissement de Transfusion Sanguine de Strasbourg, 10 rue Spielmann, BP 36, Strasbourg Cédex, 67065, France

**Keywords:** caffeine, UVc, γ, radiation, fibroblasts, cell cycle checkpoints, G2/M override

## Abstract

Caffeine has for many years been known to be involved in the sensitization of DNA to damage. One potential mechanism recently put forward is an override of the G2/M block induced by irradiation, which would leave the cells less time for DNA repair prior to mitosis. However, different cell types display a variety of responses and no clear pathway has yet emerged, especially as little is known about the capacity of this agent to enhance DNA damage in normal, untransformed cells. Continuous exposure to commonly used caffeine concentrations (1–5 mM) inhibited the proliferation of normal human fibroblasts (NHFs) in a dose-dependent manner to up to 80% at 5 mM. Exposure of exponentially growing NHFs to UVc radiation (20 J m^–2^) or γ radiation (2.5–8 Gy) led to a 45–60% inhibition of proliferation and protracted accumulation of cells in the G2/M phase. Addition of 2 mM caffeine after irradiation induced slowing of the S phase passage, with a resultant delay in G2/M accumulation mimicking a G2/M block override. These results were confirmed by stathmokinetic studies, which showed delayed entry of the cells into mitosis in the presence of caffeine. Our data demonstrate that caffeine primarily inhibits replicative DNA synthesis and suggest that, at least in normal cells, caffeine potentiates the cytotoxicity of radiation by intervening in DNA repair rather than by overriding the G2/M block. © 2000 Cancer Research Campaign


*British Journal of Cancer* (2000) **83**(3), 346–353
© 2000 Cancer Research Campaign
doi: 10.1054/ bjoc.2000.1259, available online at http://www.idealibrary.com on IDEAL®

### Caffeine does not cause override of the G2/M block induced by UVc or gamma radiation in normal human skin fibroblasts

**G Deplanque[1], F Vincent[1], MCM Mah-Becherel[1], J-P Cazenave[2], J-P Bergerat[1] and C Klein-Soyer[2]**

[1]Laboratoire d'Oncologie Moléculaire, Institut de Recherche contre les Cancers de l'Appareil Digestif, Hôpitaux Universitaires de Strasbourg, BP 426, 67091 Strasbourg, France; [2]INSERM U. 311, Etablissement de Transfusion Sanguine de Strasbourg, 10 rue Spielmann, BP 36, 67065 Strasbourg Cédex, France

**Summary** Caffeine has for many years been known to be involved in the sensitization of DNA to damage. One potential mechanism recently put forward is an override of the G2/M block induced by irradiation, which would leave the cells less time for DNA repair prior to mitosis. However, different cell types display a variety of responses and no clear pathway has yet emerged, especially as little is known about the capacity of this agent to enhance DNA damage in normal, untransformed cells. Continuous exposure to commonly used caffeine concentrations (1–5 mM) inhibited the proliferation of normal human fibroblasts (NHFs) in a dose-dependent manner to up to 80% at 5 mM. Exposure of exponentially growing NHFs to UVc radiation (20 J $m^{-2}$) or $\gamma$ radiation (2.5–8 Gy) led to a 45–60% inhibition of proliferation and protracted accumulation of cells in the G2/M phase. Addition of 2 mM caffeine after irradiation induced slowing of the S phase passage, with a resultant delay in G2/M accumulation mimicking a G2/M block override. These results were confirmed by stathmokinetic studies, which showed delayed entry of the cells into mitosis in the presence of caffeine. Our data demonstrate that caffeine primarily inhibits replicative DNA synthesis and suggest that, at least in normal cells, caffeine potentiates the cytotoxicity of radiation by intervening in DNA repair rather than by overriding the G2/M block. © 2000 Cancer Research Campaign

**Keywords**: caffeine; UVc; $\gamma$ radiation; fibroblasts; cell cycle checkpoints; G2/M override

The sensitization of cells by caffeine to radiation-induced DNA damage has been investigated for several decades (Waldren and Rasko, 1978). Pleiotropic functions have been evoked, in particular sensitization to UV or ionizing radiation or to alkylating agents, stabilization of protein complexes necessary for progression of the cell cycle (reviewed in Murnane, 1995), inhibition of clonogenic survival (Powell et al, 1995; DeFrank et al, 1996), effects on cyclin $B_1$ (Narayanan et al, 1997; Takagi et al, 1999), an increase in chromosomal aberrations (Kihlman and Odmark 1965; Ostertag et al, 1965) or induction of premature mitotic events (Schlegel et al, 1987; Downes et al, 1990). This has led to extensive research into possible mechanisms and to the now generally admitted concept that caffeine has the capacity to cause override of the G2/M block induced by irradiation (Walters et al, 1974; Rowley et al, 1984; Rowley 1992; Powell et al, 1995; DeFrank et al, 1996; Narayanan et al, 1997; Takagi et al, 1999). The multiplicity of hypotheses put forward and the variety of responses displayed by different cell types have nevertheless prevented the establishment of a clear-cut mechanism. Notably, little is know about the ability of caffeine to enhance DNA damage in normal, untransformed human cells.

The purpose of this paper was to examine the effects of caffeine on the proliferation and cell cycle kinetics of normal, untransformed cells. Normal human fibroblasts (NHFs) naturally synchronized by confluence inhibition and medium exhaustion were used either untreated or after irradiation with UVc or ionizing $\gamma$-rays.

*Received 29 November 1999*
*Revised 28 March 2000*
*Accepted 3 April 2000*

*Correspondence to*: C Klein-Soyer

### MATERIALS AND METHODS

### Materials

Cell culture media, Dulbecco's modified Eagle medium with sodium pyruvate, HEPES, phosphate buffered saline (PBS) Dulbecco's formulation, trypsin-EDTA, L-glutamine, antibiotics (penicillin and streptomycin), fungizone, fetal calf serum (FCS) (myoclone plus, virus and mycoplasma screened) and Karyo MAX® Colcemid® solution were from Gibco (Paisley, UK). Culture dishes were from Falcon, Becton-Dickinson Company (Lincoln Park, NJ, USA). Human fibronectin (FN) was purified from plasma as previously described (Ruoslahti et al, 1982). Goat anti-mouse IgG, a soluble peroxidase–anti-peroxidase complex antibody developed in mouse, 3,3′-diaminobenzidine tetrachloride, propidium iodide and ribonuclease A (Rnase A) from bovine pancreas, 5-bromo-2′-deoxyuridine (BrdU) and caffeine were from Sigma-Aldrich Corp (St Louis, MO, USA). A mouse anti-BrdU monoclonal antibody was from Caltag Laboratories (Burlingame, CA, USA). Cells were UVc-irradiated by exposure to a 254 nm 15 W UV lamp and the UV dosage was determined with a radiometer (VLX-3W, 254 nm, Bioblock, Strasbourg, France) and the cumulative exposure expressed in Joules $m^{-2}$, usually 20 J $m^{-2}$ in the present experiments. The $\gamma$ radiation source was a $^{137}$Cesium source (irradiator for blood products, IBL 437 C) delivering 6.9 Gy per min under water. All other chemicals were of analytical grade from Sigma or Merck (Darmstadt, Germany).

### Cell cultures and proliferation assay

Foreskin NHFs from a single donor were obtained following routine circumcision and cultured according to Sly and Grubb (1979) with minor modifications. The skin explants were spread on human FN (adsorbed from a 10 μg $ml^{-1}$ solution) to favour cell adhesion and migration and the culture medium was Dulbecco's modified Eagle medium containing sodium pyruvate, 10 mM HEPES, 2 mM L-glutamine, 100 U $ml^{-1}$ penicillin, 100 μg $ml^{-1}$ streptomycin, 0.25 μg $ml^{-1}$ fungizone and 10% FCS. Cell proliferation was determined in the following way. Fibroblasts synchronized in the G0/G1 phase by confluence arrest and medium exhaustion (95 ± 0.5% cells in G0/G1, mean ± SEM, $n = 20$) were seeded on FN in 100/15 mm dishes, at a concentration of $6.3 \times 10^3$ cells $cm^{-2}$ in medium containing 10% FCS, and allowed to adhere for 24 h. A representative sample was then trypsinized and counted in a Coulter ZIDT particle counter (Beckman Coulter, Gagny, France). This allowed confirmation of the efficiency of cell adhesion (96 ± 8% in control cultures, mean ± SEM, $n = 11$) and was taken as the starting count for proliferation. Caffeine was added at the required concentrations, the serum concentration was lowered to 5% and at predetermined times the numbers of cells in control and test dishes were estimated as described above. Unless stated, the medium was not changed during the proliferation assay. To allow comparison between experiments, the number of adherent cells was normalized to the culture surface area and expressed as cells $cm^{-2}$.

### Incorporation of BrdU by proliferating NHFs and blockade of cells in mitosis

Fibroblasts from post-confluent cultures were seeded at a density of $5 \times 10^3$ cells $cm^{-2}$ in 35 mm culture dishes coated with FN (0.5 μg $ml^{-1}$), in medium containing 10% FCS, and allowed to adhere for 24 h. The medium was then changed, the serum concentration lowered to 5% and caffeine added to a concentration of 0, 1, 2 or 5 mM. BrdU (10 μM) was added 16 h prior to fixation of the cells in 2% paraformaldehyde solution. Proliferating fibroblasts incorporating BrdU into the newly synthesized DNA of their nuclei were identified by an immunocytochemical technique (Labourdette et al, 1990; Klein-Soyer et al, 1992). The replicating cells displayed black nuclei after 3,3′-diaminobenzidine staining, whereas the non-replicating cells had light purple nuclei after Giemsa dye counterstaining. Cell densities were calculated by counting the nuclei in at least five calibrated random fields (0.98 $mm^2$) in each dish and results were expressed as the mean ± SEM of labelled versus total cell density.

In separate experiments, NHFs were incubated with Colcemid® (0.1 μg $ml^{-1}$) during irradiation (UVc, 20 J $m^{-2}$, or γ 2.5, 5, and 8 Gy) and caffeine treatment, in order to block the cells in mitosis and prevent their entry into a second cell cycle. At various times, 10–24 h following irradiation and addition of caffeine, representative samples were fixed, stained and counted. Results were expressed as the density of mitotic cells vs the total cell density as described above. It was observed in preliminary experiments using Colcemid® that the cells tended to detach from the substrate on accumulating in mitosis, thus preventing accurate determination of the cell density. Since this occurred within 24 h for non-irradiated NHFs but not in irradiated samples over the same period of time, no results were presented for control NHFs at 24 h.

### Cell cycle analyses

Fibroblasts from growth-arrested cultures were seeded at a density of $6.3 \times 10^3$ cells $cm^{-2}$ and grown in triplicate 100/15 mm culture dishes in medium containing 10% FCS. After 24 h, representative dishes were exposed to UVc or γ radiation. At this time, at least 50% of the cells had entered the S phase. The medium was then changed, the serum concentration lowered to 5% and 0 or 2 mM caffeine added, which constituted time zero ($t = 0$) of the experiment. Cells from the different conditions were trypsinized at various times, washed in cold PBS, fixed in 10% PBS, 90% absolute methanol and stored at –20°C until processing. An aliquot (1 ml) of thawed fixed cell suspension containing $1 \times 10^6$ cells was washed twice in cold PBS, resuspended in 500 μl cold PBS and stained with propidium iodide (final concentration 50 μg $ml^{-1}$ in the presence of 100 μg $ml^{-1}$ Rnase A) for at least 10 min in the dark on ice. The cell cycle was then analysed by flow cytometry in a FACSCalibur cell sorter (Becton Dickinson, San Jose, CA, USA) and the cell distribution in the different phases of the cycle calculated using Modfit 2.0 cell cycle analysis software.

### Statistical analyses

Results were compared by variance analysis followed by the Newman–Keuls test using the statistical software STAT-ITCF (ITCF-Boigneville, France).

### RESULTS

### Effects of caffeine on the proliferation of normal human fibroblasts

Proliferation studies were conducted using NHFs from post-confluent cultures which were seeded at low density and allowed to adhere for 24 h. At this time, caffeine was added at a final concentration of 0, 1, 2 or 5 mM and the serum concentration was lowered to 5% FCS to optimize drug-induced responses. In control cultures, the cell doubling or generation time (Klein-Soyer et al, 1997) under these conditions was 23 ± 1.5 h (mean ± SEM, $n = 16$). Continuous exposure of NHFs to caffeine for 72 h led to a dose-dependent inhibition of proliferation (Figure 1A). At the highest concentration of caffeine (5 mM), cell replication was almost totally inhibited and the final cell density at 72 h was less than 20% of the control density. BrdU labelling demonstrated that the proportion of nuclei synthesizing DNA was over 90% for the first 24 h even in the presence of 5 mM caffeine, but then started to decrease as a function of caffeine concentration, reaching 17 ± 2% for 5 mM caffeine at 64 h, although the cell density remained very low (not more than twice that at time zero) (Figure 1A, panels a–d). In controls, BrdU labelling was strong up to 48 h and then diminished rapidly as the cells reached confluence (over 8-fold the density at time zero) (Figure 1A, panel a). In one series of dishes, where medium containing caffeine (5 mM) was removed after 48 h and replaced by medium without caffeine, the inhibitory effect of caffeine was reversed and BrdU incorporation started to increase, reaching 46 ± 4% at 64 h and 73 ± 7% at 96 h (Figure 1B, panel f). Consequently, the cell density also increased (13 400 ± 1140 cells $cm^{-2}$ after removal of caffeine versus 8700 ± 873 cells $cm^{-2}$ when 5 mM caffeine was maintained). These results were confirmed by cells cycle analysis experiments, in which 53% of

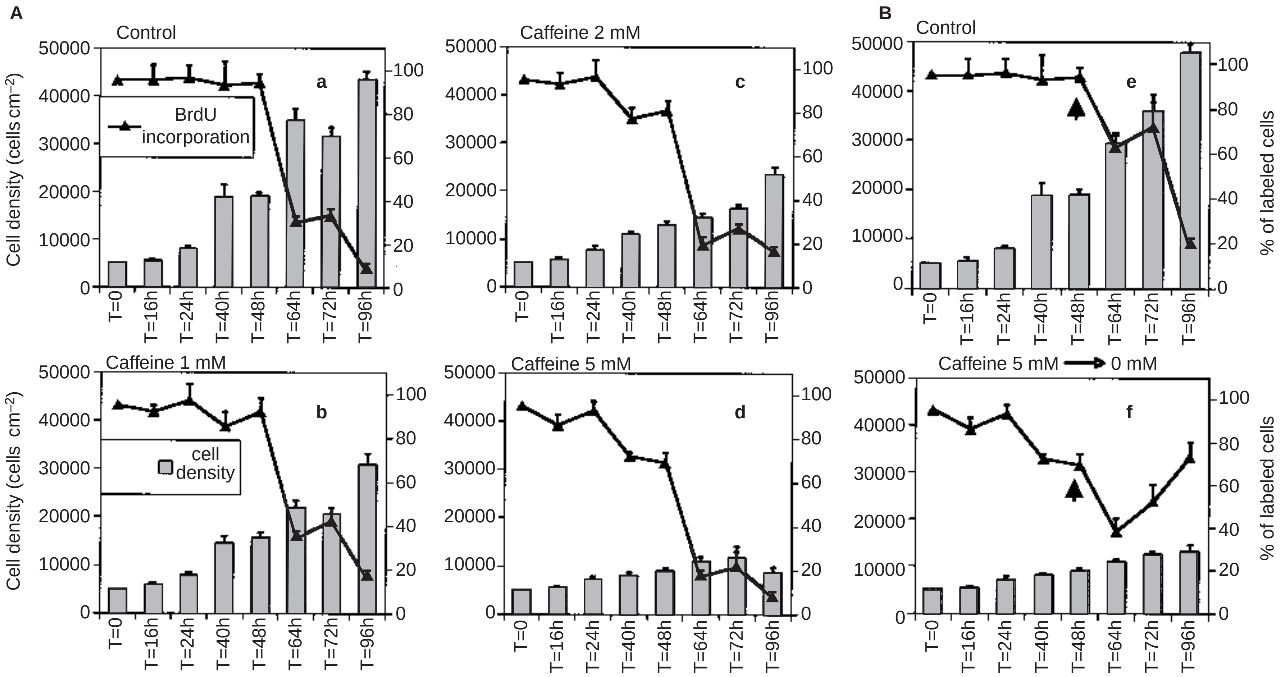

**Figure 1** Effect of increasing concentrations of caffeine on the proliferation and BrdU incorporation of NHFs. Sparsely seeded NHFs were grown in the presence of caffeine (1, 2 and 5 mM) and BrdU incorporation was estimated as described in Materials and methods. (**A**) Continuous exposure to caffeine. Results for cell proliferation are representative of at least three experiments. (**B**) At 48 h (arrow), the medium was replaced by medium without caffeine after extensive washing of the cultures. Results are from one experiment performed in duplicate the cells had entered the S phase at 72 h, 24 h after removal of caffeine, whereas in cultures continuously exposed to 5 mM caffeine, 83% of the cells remained in the G0/G1 phase and only 9% had entered the S phase.

### Caffeine and radiation have additive inhibitory effects on the proliferation of NHFs

NHFs from post-confluent cultures (G0/G1 ≈ 95%) were seeded as in cell proliferation experiments. After 24 h, when over 50% of the cells were in the S phase (*S*: 59 ± 5%, G2/M: 8 ± 2%, mean ± SEM, *n* = 7), representative samples were irradiated with UVc or γ-rays and caffeine was added immediately and maintained throughout the experiment. UVc radiation (20 J $m^{-2}$) (Figure 2A), γ radiation (2.5, 5 or 8 Gy) (Figure 2B) or 2 mM caffeine significantly inhibited NHF proliferation as a function of time, and the inhibitory effect of caffeine was additive with that induced by either type of radiation. Subsequently, the cell cycle kinetics were investigated under identical conditions.

### Effects of caffeine on the progression of irradiated NHFs through the cell cycle

Cell cycle kinetics were analysed after irradiating exponentially growing NHFs with either UVc (20 J $m^{-2}$ or γ radiation (2.5, 5 or 8 Gy) when over 50% of the cells were in the S phase (62 ± 3%, mean ± SEM, *n* = 14). Caffeine (2 mM) was added immediately after irradiation and maintained throughout the experiment. In the presence of caffeine, NHFs slowly completed the ongoing cycle before entering a new cycle where cells accumulated in the G1 phase. This was demonstrated by the inhibition of cells proliferation as compared to the exponential proliferation of control cultures (Figure 3, panels a and b).

As expected, UVc radiation retarded the cycle by provoking a transient accumulation of NHFs in the G2/M phase (50.3 ± 2.7% at 16 h, mean ± SEM, *n* = 3), after which the cells continued to proliferate (Figure 3, panel c). When caffeine was added to irradiated NHFs, the inhibition of proliferation was almost total and only 25.9 ± 6% of the cells had entered the G2/M phase at 16 h (mean ± SEM, *n* = 3, $P < 0.05$) while 56.7 ± 0.8% remained in the S-phase (mean ± SEM, *n* = 3) (Figure 3, panel d). Hence, the lesser accumulation of cells in the G2/M phase 16 h following UVc and caffeine treatment was clearly due to a delay in passage through the S phase. In addition, at 48 and 72 h, G2/M phase levels were even higher in caffeine-treated cells as compared to controls. The concomitant lack of cell proliferation, as shown by cell density measurements, was again consistent with the absence of a G2/M block override. Similarly, using γ radiation (2.5, 5 or 8 Gy) and 2 mM caffeine, the presence of caffeine retarded the entry of cells into the G2/M phase at all radiation doses employed. The delay in the accumulation of irradiated cells in the G2/M phase varied from 10 h (2.5 Gy) to 24 h (5 and 8 Gy). Whereas this accumulation reached 66.9 ± 3.5% in irradiated control NHFs (mean ± SEM, *n* = 4), in caffeine-treated irradiated NHFs, the percentage of cells in the G2/M phase attained only 31.2 ± 5% (mean ± SEM, *n* = 4, $P < 0.05$). Thereafter the rate of cell proliferation remained significantly lower than in cultures submitted to radiation alone. Representative results for the dose of 2.5 Gy are presented in Figure 4. In irradiated control NHFs (panel c), the important accumulation of cells in the G2/M phase reached 64% at 10 h and only 41% in the presence of caffeine (panel d). After 24 h the irradiated control cells started to proliferate again as indicated by the increase in cell density.

© 2000 Cancer Research Campaign

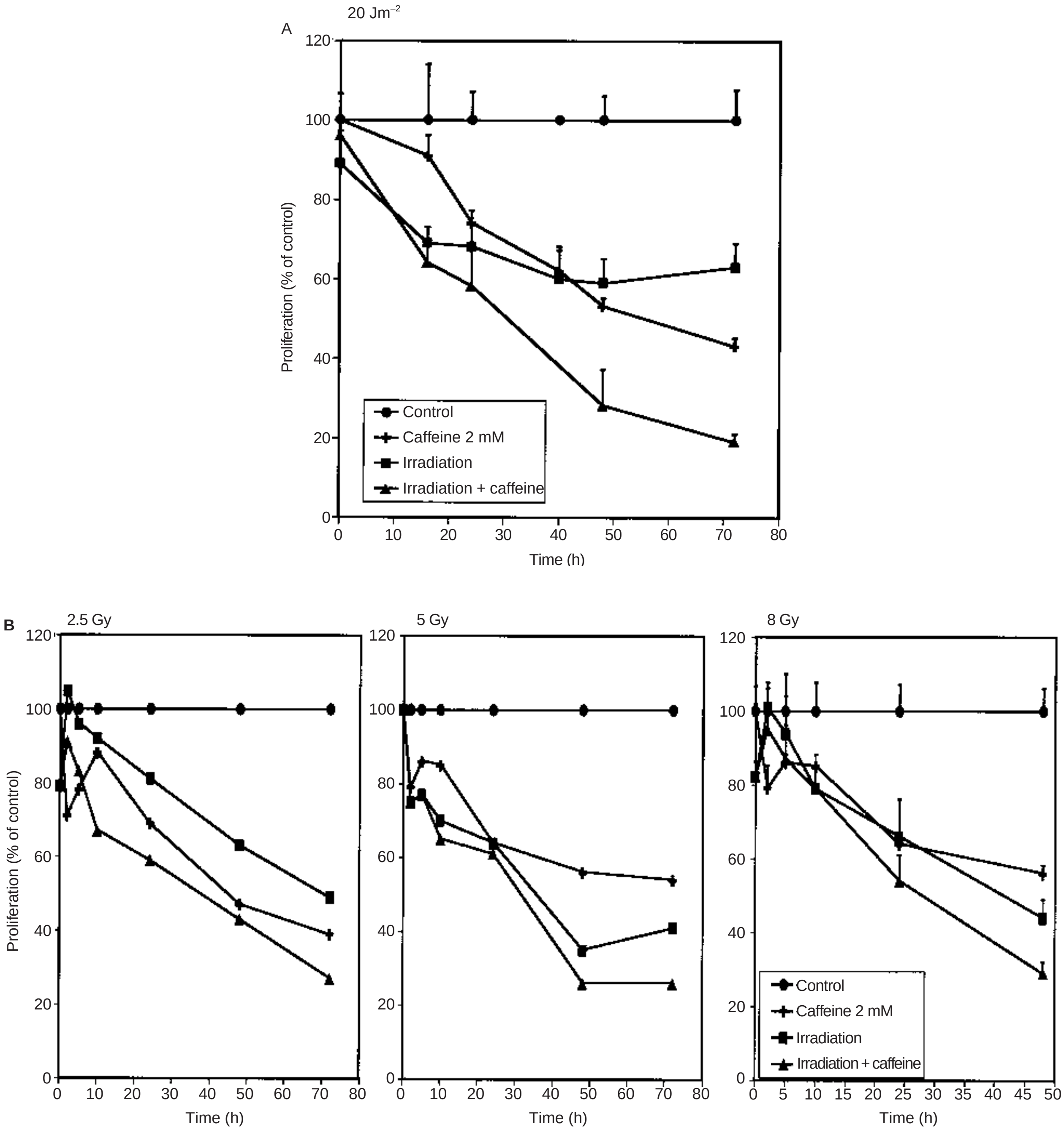

**Figure 2** Effects of caffeine on the proliferation of irradiated NHFs. NHFs were cultured as described in Materials and methods and at the time of irradiation the serum concentration was lowered to 5% FCS and 2 mM caffeine was added. Results were calculated from absolute cell densities in treated and control cultures at each time point. (**A**) NHFs submitted to UVc radiation (20 J $m^{-2}$). Results are expressed as the percentage of the control value set to 100% and are the mean ± SEM of three separate experiments performed in triplicate. (**B**) NHFs submitted to $\gamma$ radiation (2.5, 5 or 8 Gy). Results are expressed as the percentage of the control value set to 100% and are the mean of triplicates (2.5 and 5 Gy), or for 8 Gy the mean ± SEM of two separate experiments performed in triplicate

In separate assays, experiments were performed 6 h after seeding when the cells were still in the G0/G1 phase (97 ± 1%, mean ± SEM, $n$ = 3). In control cultures, NHFs entered the S phase after 16 h and then proliferated exponentially before accumulating in the G0/G1 phase as a result of confluence and medium exhaustion (Figure 5, panel a). Addition of 2 mM caffeine caused NHFs to progress slowly through one cell cycle and accumulate in the G0/G1 phase after a single replication (Figure 5, panel b). Exposure of the cells to UVc radiation (20 J $m^{-2}$) retarded their release from the G0/G1 phase for up to 40 h, after which they proliferated normally (Figure 5, panel c). Finally, addition of caffeine to irradiated cells significantly hindered their entry into the S phase and at 96 h only 28% of the cells had left the G0/G1 phase (Figure 5, panel d).

*© 2000 Cancer Research Campaign* 

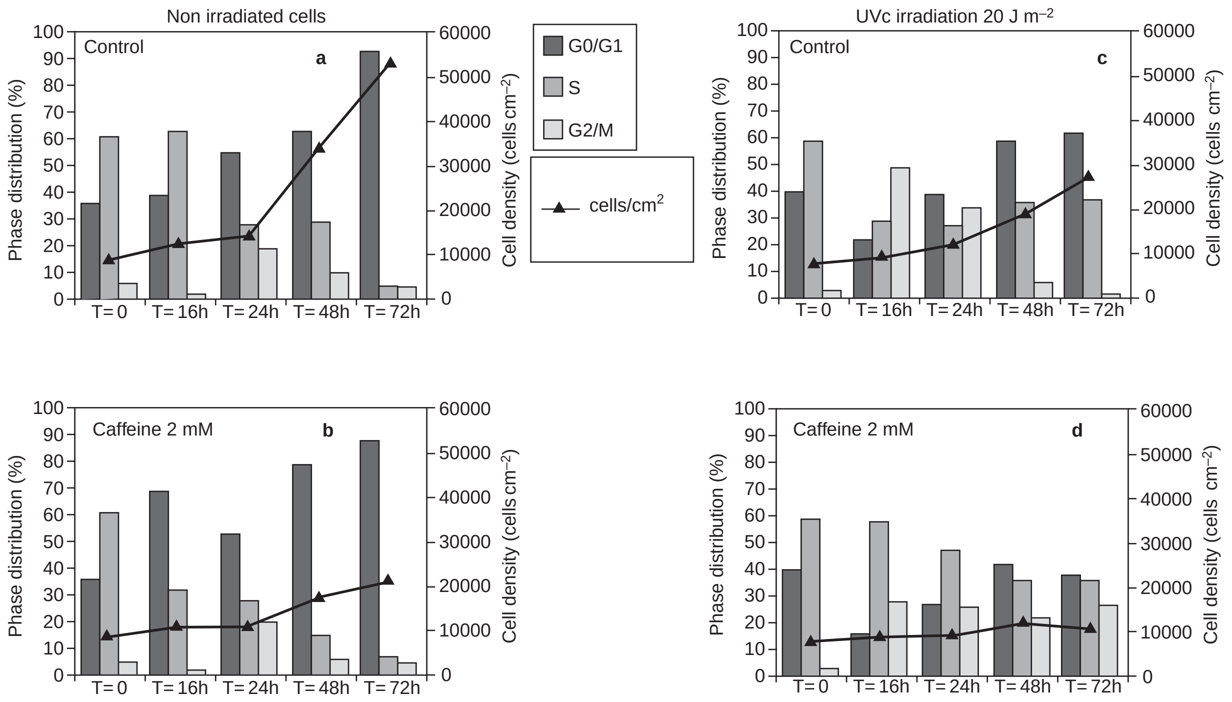

**Figure 3** Effects of caffeine on the cell cycle kinetics of exponentially growing NHFs submitted to UVc radiation. Experiments were performed as described in Materials and methods and the cell phase distribution and corresponding cell density are represented as a function of time. One experiment representative of three

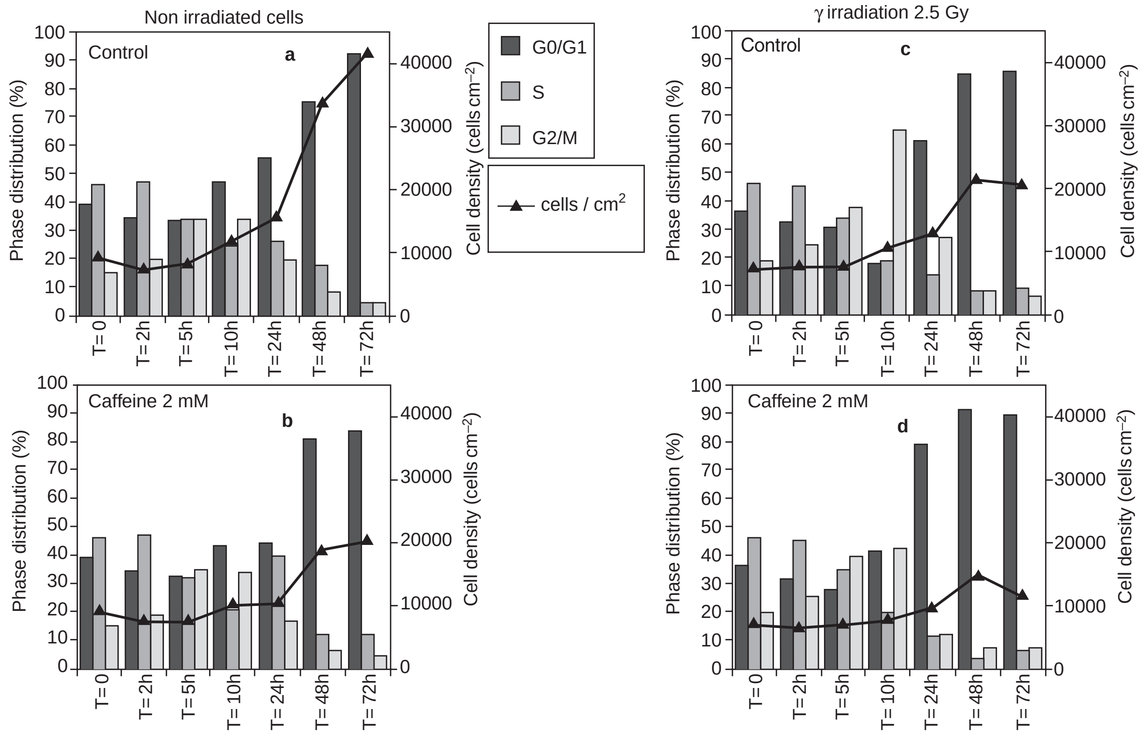

**Figure 4** Effects of caffeine on the cell cycle kinetics of exponentially growing NHFs submitted to γ radiation. Experiments were performed as described in Materials and methods and the cell phase distribution and corresponding cell density are represented as a function of time

*© 2000 Cancer Research Campaign*

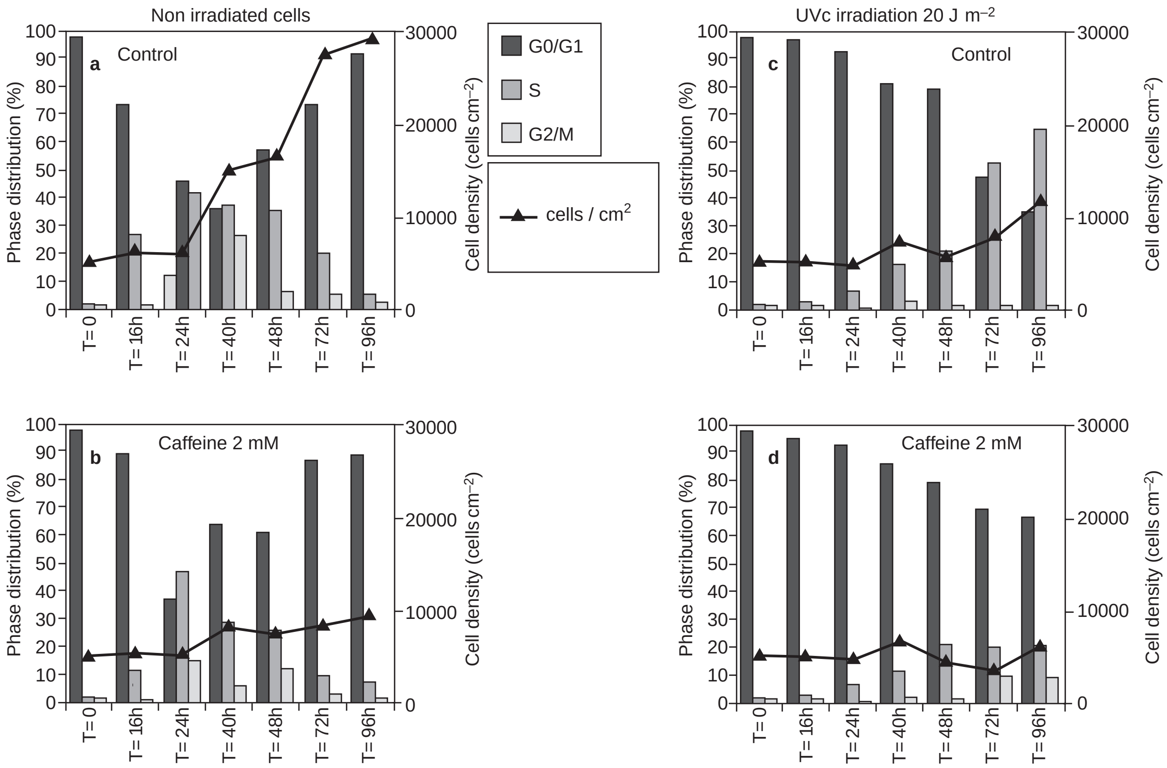

**Figure 5** Effects of caffeine on the cell cycle kinetics of NHFs synchronized in the G0/G1 phase and submitted to UVc radiation. Experiments were carried out as described in Figure 4 except that the cells were irradiated 6 h after seeding. One experiment representative of three

### Delay of Colcemid® arrested mitosis in caffeine treated NHFs

In order to confirm that caffeine itself does not facilitate the passage of NHFs through the G2 phase, its effects were analysed in stathmokinetic experiments early during the ongoing cell cycle, in the presence of Colcemid® to prevent the cells entering a second cycle. NHF cultures with over 75% of the cells in the S phase were used for this assay. In non-irradiated cultures, after 10 h in the presence of Colcemid®, 42 ± 5% of the cells had accumulated in mitosis in the absence of caffeine, as compared to only 9 ± 1% in its presence ($P < 0.05$). At this time, in irradiated cultures only cells treated with 2.5 Gy γ-rays had started to accumulate in mitosis and less rapidly when caffeine was present (Table 1). Fourteen hours later, at time 24 h, 29 ± 3% of UVc irradiated cells were blocked in mitosis in the absence of caffeine and 13 ± 2% in its presence ($P < 0.05$). In γ-irradiated cultures, the accumulation of cells in mitosis was at this time inversely proportional to the radiation dose and consistently lower in the presence of caffeine (Table 1).

### DISCUSSION

In the present paper, we demonstrate that caffeine induces a dose-dependent inhibition of the proliferation of diploid NHFs, at least at doses currently used in the literature, and that this inhibitory effect is reversible within 24 h of removal of caffeine. As expected, UVc or γ-irradiation of exponentially growing NHFs led to a transient accumulation of cells in the G2/M phase. Addition of caffeine under the same conditions slowed the progression of cells through the S phase and retarded their entry into the G2/M phase, thus mimicking an override. These results were confirmed by stathmokinetic experiments in which caffeine delayed the accumulation of mitotic cells. Therefore, caffeine does not cause override of the G2/M block but inhibits progression through the cell cycle at the G0/G1 and S phases, thus delaying the arrival of cells in the G2/M phase.

Caffeine has been known for a long time to inhibit cell proliferation, as for instance that of HeLa, CHO and 3T3 fibroblast cells (Ostertag et al, 1965; Walters et al, 1974; Levi-Shaffer and Touitou, 1991). Although the diversity of the available data is somewhat confusing, the general indication is that caffeine inhibits both cell proliferation and DNA repair by interacting with synthesis/repair polymerases. However, analyses to data of the effects of caffeine on the cell cycle of irradiated cells have led to the unanimous conclusion of the facilitation of cell cycle progression and an override of the radiation-induced G2/M block (Walters et al, 1974; Rowley et al, 1984; Rowley, 1992; Powell et al, 1995; DeFrank et al, 1996; Narayanan et al, 1997; Takagi et al, 1999). The discrepancy between these two concepts, namely an inhibition of cell proliferation but the facilitation of cell cycle progression, has not, to our knowledge, been approached previously.

In order to elucidate this point, we studied the effects of caffeine on diploid NHFs either untreated or submitted to irradiation. As the sensitivity to DNA-damaging agents depends strongly on the proliferation status of the cells (reviewed in Kaufmann and Paules, 1996), many cell cycle studies have been performed using the mitotic shake technique (Walters et al, 1974; Rowley et al, 1984) or chemically synchronized cells (Rowley et al, 1984; Orren et al, 1995; Narayanan et al, 1997). Although selective, these methods also have some disadvantages. Thus, mitotic shake selection does

© 2000 Cancer Research Campaign 

**Table 1** Percentage of Colcemid®-induced mitoses in NHF cultures at different times following UVc or $\gamma$ irradiation and addition of caffeine

| Time after treatment | | Colcemid® 0.1 $\mu$g ml$^{-1}$ | Caffeine + Colcemid® |
|---|---|---|---|
| 10 h | Non-irradiated | 42 ± 5[a] | 9 ± 1 |
| | Irradiated UVc, 20 J m$^{-2}$ | 2 ± 1 | 1 ± 0 |
| | $\gamma$ 2.5 Gy | 7 ± 2 | 4 ± 1 |
| | $\gamma$ 5 Gy | 1 ± 0 | 1 ± 0 |
| | $\gamma$ 8 Gy | 1 ± 0 | 0 ± 0 |
| 24 h | Irradiated UVc, 20 J m$^{-2}$ | 29 ± 1[a] | 13 ± 2 |
| | $\gamma$ 2.5 Gy | 34 ± 5 | 26 ± 3 |
| | $\gamma$ 5 Gy | 20 ± 4[a] | 8 ± 1 |
| | $\gamma$ 8 Gy | 12 ± 2[a] | 5 ± 1 |

Results are the mean ± SEM from three separate experiments performed in duplicate. Percentages of cells blocked in mitosis were quantified as described in Materials and methods. [a]Significantly different from NHFs treated with caffeine and Colcemid®, $P < 0.05$

not discriminate between live and dead cells or cell debris, while chemical synchronization can induce unexpected side-effects which lead to misinterpretation of the results (Ji et al, 1997). Hence we carried out the present experiments using NHFs synchronized naturally by confluence and medium exhaustion, in accordance with previously determined conditions (Kaufmann and Wilson, 1990; Dulic et al, 1994).

NHFs from post-confluent cultures seeded at low density were over 95% in the G1 phase 6 h after seeding and 60–80% in the S phase 24 h after seeding. Caffeine retarded the release of non-irradiated cells from the G0/G1 phase and their progression through the S phase (Figure 4, panel b and Figure 5, panel b). As shown by the parallel cell cycle and cell proliferation kinetics, the presence of caffeine caused NHFs to traverse the ongoing cell cycle and then accumulate in the G0/G1 phase. This effect was reversible even after 48 h exposure to 5 mM caffeine within 24 h of its removal: BrdU incorporation and the S phase population increased to 70% and 53% respectively, as compared to 10% and 9% in NHFs still exposed to caffeine.

UVc or $\gamma$ radiation induced a transient G2/M block in exponentially proliferating NHFs soon after irradiation. In the presence of caffeine, the proportion of cells in the G2/M phase at this time was systematically lower, perfectly mimicking a G2/M block override when cell proliferation was neglected. On the other hand, an overall analysis of cell cycle kinetics and cell proliferation led to the conclusion that addition of caffeine to NHFs irradiated in the exponential phase slowed their progression through the cell cycle. Consequently, it retarded the entry of cells into the G2/M phase but in no circumstances allowed override of the block. Caffeine also retarded release from the G0/G1 phase when NHFs were irradiated at this stage. A definitive argument was provided by stathmokinetic studies, in which caffeine systematically delayed the accumulation of mitotic cells in both non-irradiated and irradiated cultures. This is just the contrary of what one would expect if caffeine facilitated the G2/M transition. Our present data apply to normal diploid fibroblasts and we are currently investigating whether identical results can be obtained in transformed or tumoural cell lines. Preliminary experiments performed on the transformed Chinese ovary CHO-K1 cell line have led to the same conclusion. These results demonstrate the importance of performing cell cycle analyses and cell proliferation assays in parallel, and while confirming that caffeine has inhibitory effects additive with those of irradiation, show that caffeine interacts with both UVc and ionizing radiation by inhibiting progression through the cell cycle at the G0/G1 and S phases and not by shortening the G2/M block.

### ACKNOWLEDGEMENTS

The authors would like to thank Mrs Marlène Ehret and Francine Noël for excellent technical assistance, the Service de Chirurgie Infantile des Hôpitaux Universitaires de Strasbourg for providing human foreskin samples and Miss Juliette Mulvihill for reviewing the English of the manuscript. Part of this work was supported by the Association pour la Thérapie Génique des Cancers (ATGC). Part of this work was presented at the 90th annual meeting of the American Association for Cancer Research, April 10–14 1999, Philadelphia, and published in abstract form (*Proceedings of the AACR* 1999; **40** (Abstract 951): 143).

### REFERENCES

DeFrank JS, Tang W and Powell SN (1996) p53-null cells are more sensitive to ultraviolet light only in the presence of caffeine. *Cancer Res* **56**: 5356–5368

Downes CS, Musk SRR, Watson JV and Johnson RT (1990) Caffeine overcomes a restriction point associated with DNA replication, but does not accelerate mitosis. *J Cell Biol* **110**: 1855–1859

Dulic V, Kaufmann WK, Wilson SJ, Tisty TD, Lees E, Harper WJ, Elledge SJ and Reed SI (1994) p53-dependent inhibition of cyclin-dependent kinase activities in human fibroblasts during radiation-induced G1 arrest. *Cell* **76**: 1013–1023

Ji C, Marnett LJ and Pietenpol JA (1997) Cell cycle re-entry following chemically-induced cell cycle synchronization leads to elevated p53 and p21 protein levels. *Oncogene* **15**: 2749–2753

Kaufmann WK and Paules RS (1996) DNA damage and cell cycle checkpoints. *FASEB J* **10**: 238–247

Kaufmann WK and Wilson SJ (1990) DNA repair endonuclease activity during synchronous growth of diploid human fibroblasts. *Mutat Res* **236**: 107–117

Kihlman BA and Odmark G (1965) Deoxyribonucleic acid synthesis and the production of chromosomal aberrations by streptonigrin, 8-ethoxy-caffeine and 1,3,7,9-tetramethyluric acid. *Mutat Res* **2**: 494–505

Klein-Soyer C, Archipoff G, Beretz A and Cazenave J-P (1992) Opposing effects of heparin with TGF-β or aFGF during repair of a mechanical wound of human endothelium. Influence of cAMP on cell migration. *Biol Cell* **75**: 155–162

Klein-Soyer C, Céraline J, Orvain C, de la Salle C, Bergerat J-P and Cazenave J-P (1997) Angiogenesis inhibitor SR 25989 upregulates thrombospondin-1 expression in human vascular endothelial cells. *Biol Cell* **89**: 295–307

Labourdette G, Janet T, Laeng P, Perraud F, Lawrence D and Pettman B (1990) Transforming growth factor-β modulates the effects of basic fibroblast growth factor on growth and phenotypic expression of rat astroblast in vitro. *J Cell Physiol* **44**: 473–484

Levi-Schaffer F and Touitou E (1991) Xanthines inhibit 3T3 fibroblast proliferation. *Skin Pharmacol* **4**: 286–290

Murnane JP (1995) Cell cycle regulation in response to DNA damage in mammalian cell: A historical perspective. *Cancer Metastasis Rev* **14**: 17–29

Narayanan PK, Rudnick JM, Walthers EA and Crissman HA (1997) Modulation in cell cycle and cyclin B1 expression in irradiated HeLa cells and normal human skin fibroblasts treated with staurosporine and caffeine. *Exp Cell Res* **233**: 118–127

Orren DK, Petersen LN and Bohr VA (1995) A UV-responsive G2 checkpoint in rodent cells. *Mol Cell Biol* **15**: 3722–3730

Ostertag W, Duisberg E and Stürmann M (1965) The mutagenic activity of caffeine in man. *Mutat Res* **2**: 293–296

Powell SN, DeFrank JS, Connel P, Eogan M, Preffer F, Dombkovski D, Tang W and Friend S (1995) Differential sensitivity of p53$^{(-)}$ and p53$^{(+)}$ cells to caffeine-induced radiosensitization and override of $G_2$ delay. *Cancer Res* **55**: 1643–1648

 *© 2000 Cancer Research Campaign*

Rowley R (1992) Reduction of radiation-induced $G_2$ arrest by caffeine. *Radiat Res* **129**: 224–227

Rowley R, Zorch M and Leeper DB (1984) Effect of caffeine on radiation-induced mitotic delay: Delayed expression of $G_2$ arrest. *Radiat Res* **97**: 178–185

Ruoslahti E, Haymann EG, Perschbacher M and Engvall E (1982) Fibronectin: purification, immunochemical properties and biological activities. *Methods Enzymol* **82**: 803–831

Schlegel R, Croy RG and Pardee AB (1987) Exposure to caffeine and suppression of DNA replication combine to stabilize the proteins and RNA required for premature mitotic events. *J Cell Physiol* **131**: 85–91

Sly WS and Grubb J (1979) Isolation of fibroblasts from patients. *Methods Enzymol* **48**: 444–450

Takagi M, Shigeta T, Asada M, Iwata S, Nakazawa S, Kanke Y, Ishimoto K and Mizutani S (1999) DNA damage-associated cell cycle and cell death control is differentially modulated by caffeine in clones with p53 mutations. *Leukemia* **13**: 70–77

Waldren CA and Rasko I (1978) Caffeine enhancement of X-ray killing in cultured human and rodent cells. *Radiat Res* **73**: 95–110

Walters RA, Gurley LR and Tobey RA (1974) Effect of caffeine on radiation-induced phenomena associated with cell-cycle traverse of mammalian cells. *Biophys J* **14**: 99–118

© 2000 Cancer Research Campaign
